# Isolated Meckel’s diverticulum perforation as a sequel to blunt abdominal trauma: a case report

**DOI:** 10.1186/1752-1947-8-111

**Published:** 2014-04-02

**Authors:** Christopher N Ekwunife, Tobechi N Mbadugha, Udonna N Ogbue

**Affiliations:** 1 Department of Surgery, Federal Medical Centre, P.M.B 1010 Owerri, Nigeria

**Keywords:** Abdominal trauma, Meckel’s diverticulum, Perforation

## Abstract

**Introduction:**

Meckel’s diverticulum is the commonest congenital abnormality of the gastrointestinal tract. Its infrequent occurrence is mirrored by the paucity of large series of data on it in the literature. Hemorrhage, obstruction and inflammation are the three main categories of complications resulting from Meckel’s diverticulum. Perforation of Meckel’s diverticulum following blunt abdominal injury is very rare indeed. We present what we believe to be the first case to be published from Africa.

**Case presentation:**

A 29-year-old Nigerian Igbo man presented with progressively worsening abdominal pain following a road traffic accident. He was a front-seat passenger traveling without a seat belt. On physical examination his abdomen was distended with guarding and rigidity. A provisional diagnosis of peritonitis secondary to perforation of intestinal viscus was made. Our patient had an emergency laparotomy, where a perforated Meckel’s diverticulum was discovered. A segmental resection of his ileum and reanastomosis were done. He had postoperative surgical site infection, but was asymptomatic for three months of follow-up.

**Conclusion:**

Perforation of Meckel’s diverticulum is rarely suspected as a cause of peritonitis following blunt abdominal injury. This case indicates the need to be aware of the possibility to limit morbidity associated with delayed management of such a perforation.

## Introduction

Meckel’s diverticulum is a congenital true diverticulum of the distal ileum. The German anatomist Johann Friedrich Meckel was first to describe its embryological and pathological features in 1809. Although it is the commonest congenital abnormality of the gastrointestinal tract, its infrequent occurrence is mirrored by the fact that most publications describing it are case reports or small series of cases. It has a reported incidence of 1% to 2% with a lifetime complication rate of 4% [1,2]. These complications fall into three main categories: hemorrhage, obstruction and inflammation [3]. Factors associated with increased risk of complications include male sex, age below 50 years, presence of heterotopic mucosa within the diverticulum, length of diverticulum greater than 2cm, or a diverticulum height to diameter ratio of greater than two [1,4,5]. Perforation of the diverticulum following blunt abdominal trauma is a very rare occurrence.

## Case presentation

A 29-year-old Nigerian Igbo man presented to the accident and emergency department of our hospital with an 11-hour history of worsening abdominal pain that started around the umbilicus and later became generalized. He had been a front-seat passenger in a vehicle that had burst a tire and subsequently hit a tree. He had not been wearing a seat belt, and his chest and abdomen had hit the dashboard. He had transient concussion and started vomiting after presenting to our hospital. On physical examination, he was pale, with a blood pressure of 100/80mmHg and a pulse rate of 120 beats/min. His abdomen, which was bruised on the left hypochondrium and iliac fossa, was distended and rigid.

**Figure 1:**
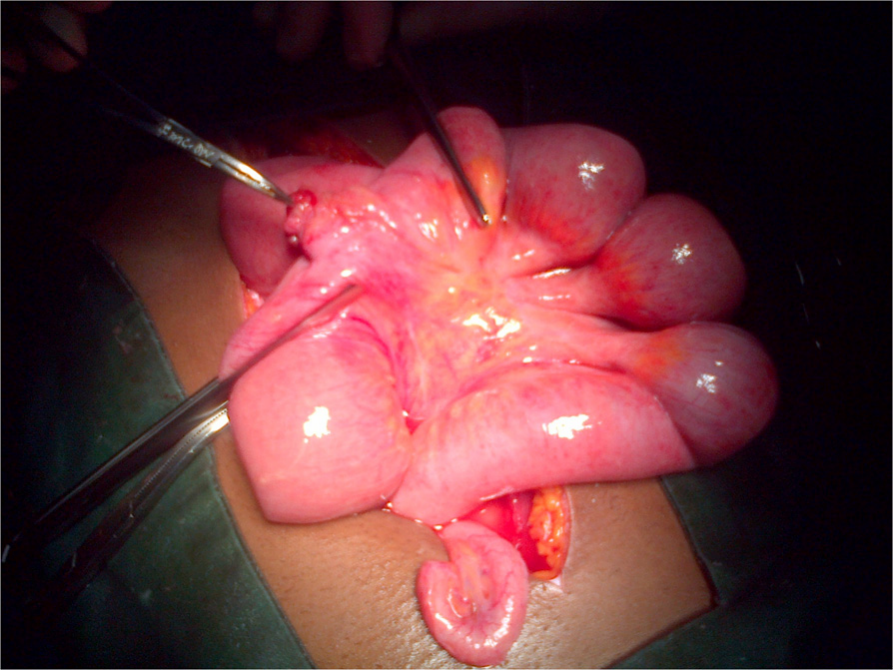
**Intraoperative view of the perforated Meckel’s diverticulum.** The hemostat is pointing to the perforation.

**Figure 2:**
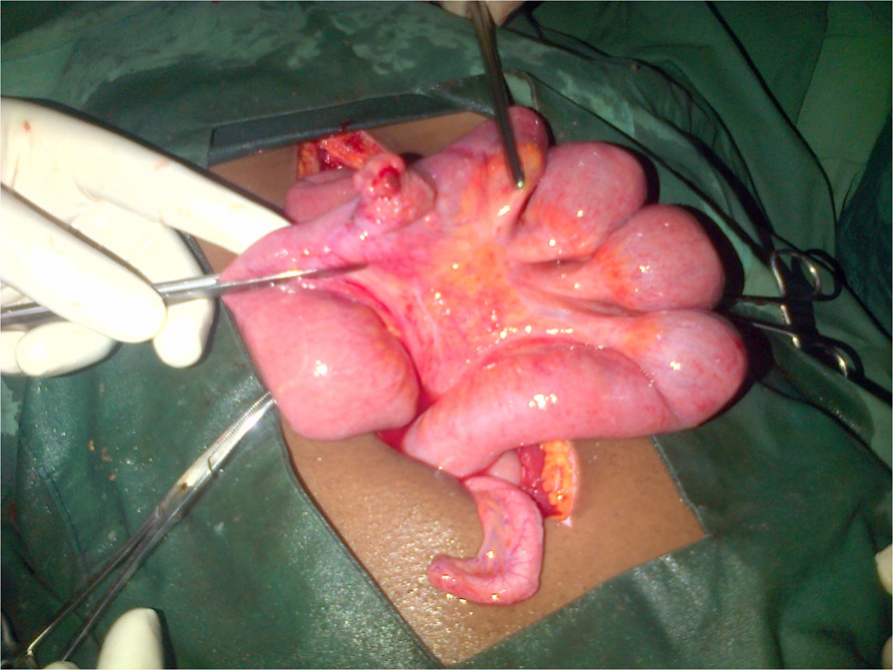
Another intraoperative view of the perforated Meckel’s diverticulum.

Paracentesis yielded straw-colored fluid mixed with blood. A radiological investigation revealed a transverse fracture of his left femur. He was resuscitated with intravenous fluids and antibiotics. With a preoperative diagnosis of peritonitis, believed to probably be due to small bowel perforation, he was taken to our operating room. On exploration, 950mL of feculent fluid mixed with blood was aspirated from his peritoneal cavity. A perforated Meckel’s diverticulum was identified 60cm from his ileocecal junction. It had a height of 5cm and a base of 3cm (Figures 1 and 2). A segmental ileal resection including the Meckel’s diverticulum was performed.

After the operation, our patient developed a superficial surgical site infection. Histopathological examination of the specimen showed no heterotopic epithelium, but there was expansion of the Peyer’s patches and lymphocytic cell infiltration of the submucosa. Ten days after the surgery, our patient had an intramedullary nailing for the femoral fracture. After three months of follow-up, he has been quite healthy.

## Discussion

Various nonspecific symptoms have been ascribed to Meckel’s diverticulum, but only 16% of patients may be symptomatic [1]. Preoperative diagnosis has been described to be as low as 5.7%, but this has improved with the use of technetium 99m pertechnetate scans [6]. In a patient presenting with acute abdomen following blunt abdominal trauma, attention is usually drawn to the more probable causes like injuries to the spleen and liver. Where perforation of hollow viscus is suspected, interest is usually drawn to the jejuno-ileum.

Perforation of Meckel’s diverticulum more commonly results from progressive diverticulitis. Less commonly, foreign bodies have been implicated in its perforation. Rarely, cases of perforation following blunt abdominal trauma have been reported, the first being by Park and Lucas in 1970 [7]. Four such cases have been reported in the medical literature [8-10]. We believe that our report is the first from Africa.

It would be difficult to decipher a pattern to this form of injury from the few reports available. Seat-belt use is associated with increased incidence of small bowel injuries; Kazemi *et al*. [10] attributed seat-belt use to Meckel’s diverticulum perforation. However, our patient was not wearing a seat belt. There was also no heterotopic epithelium in the diverticulum, something that has been associated with increased incidence of complications. The diverticulum was also relatively short, with a height to diameter ratio of less than two. However, our patient was male and less than 50 years old, factors that account for greater risk of complications in a diverticulum [4,5]. It is probable that an underlying inflammation of the Meckel’s diverticulum, underscored by the abundant Peyer’s patches and lymphocytes, facilitated its perforation by the force of the trauma.

Preoperative diagnosis of perforated Meckel’s diverticulum is a major challenge, more so in our environment where computed tomography is not readily available. Clinical signs of peritonitis with or without radiological evidence of air under the diaphragm will usually lead to a proximate diagnosis of small bowel perforation. A more specific diagnosis, however, will lead to greater recourse to a laparoscopic approach in its treatment [11].

## Conclusion

Perforation of Meckel’s diverticulum is rarely suspected as a cause of peritonitis following blunt abdominal injury. This case indicates the need to be aware of the possibility to limit the morbidity associated with delayed management of such a perforation.

## Consent

Written informed consent was obtained from the patient for publication of this case report and accompanying images. A copy of the written consent is available for review by the Editor-in-Chief of this journal.

## Competing interests

The authors declare that they have no competing interests.

## Authors’ contribution

CNE prepared the manuscript. TNM did the literature review. All authors have read and approved the manuscript.
